# Usage of procalcitonin and sCD14-ST as diagnostic markers for postoperative spinal infection

**DOI:** 10.1186/s10195-022-00644-9

**Published:** 2022-06-01

**Authors:** Xi Zhu, Kaige Li, Jianping Zheng, Gen Xia, Feng Jiang, Huan Liu, Jiandang Shi

**Affiliations:** 1 Orthopedics, General Hospital of Ningxia Medical University, Yinchuan, China; 2 Department of Surgery, School of Clinical Medicine, Ningxia Medical University, Yinchuan, China; 3 Department of Medicine, School of Medicine and Dentistry, Aab Cardiovascular Research Institute, University of Rochester, Rochester, NY USA

**Keywords:** Procalcitonin, Soluble CD14 subtype, C-reactive protein, Spinal surgery, Postoperative infection, Postoperative non-infection

## Abstract

**Objective:**

Identifying biomarkers for early diagnosis of postoperative spinal infection is essential to avoid complications after spine surgery. The presented study evaluated serum levels of procalcitonin (PCT), C-reactive protein (CRP), and soluble CD14 subtype (sCD14-ST) in patients who underwent spinal surgery to assess the diagnosis values of PCT and sCD14-ST.

**Methods:**

Serum levels of PCT, CRP, and sCD14-ST were measured in 490 (289 male/201 female) patients who underwent spinal surgery (SS) before and 1 day after surgery. PCT and sCD14-ST levels of patients diagnosed with postoperative infection (PI) and patients diagnosed with postoperative non-infection (PN) were compared.

**Results:**

Serum levels of PCT, CRP, and sCD14-ST were significantly increased after surgery (*F* = 58.393, *P* = 0.000). In patients diagnosed as having a PI, serum levels of PCT and sCD14-ST were positively correlated with each other (*r* = 0.90, *P* < 0.01) and with operation duration (*r* = 0.92, 0.88, *P* < 0.01). Receiver operating characteristic (ROC) models showed that both PCT (AUC = 0.817, optimal cutoff: 0.69 ng/ml, *P* = 0.000) and sCD14-ST (AUC = 0.824, optimal cutoff: 258.27 pg/ml, *P* = 0.000) can distinguish PI versus PN patients well.

**Conclusion:**

Our results demonstrated that serum levels of PCT and sCD14-ST have the potential to be used as a diagnostic markers for postoperative spinal infection.

## Introduction

Postoperative infection following spine surgery can be a particularly devastating complication in both the short term and the long term; it requires prolonged treatment and is associated with increased morbidity and poor outcomes for patients [1]. Postoperative infections are primarily caused by Gram-positive cocci such as* Staphylococcus aureus*, which is the most commonly reported pathogen. Patients diagnosed with postoperative infection often suffer a high risk of pseudoarthrosis, chronic pain, and adverse neurological sequelae [2–7]. More severely, deep infections after spine surgery can even cause death [8]. The incidence of postoperative spinal infection has been reported in the literature to be between 0.5 and 18.8%, while the incidence of deep infection after instrumented spinal surgery remains between 0.4 and 4.3% [2, 7, 9–12]. The variation of the incidence of postoperative infection is in part due to variations in spine surgery. The incidence for patients undergoing fusion after spinal trauma has been reported to be higher than average [13]. The frequent incidence and destructive impact of postoperative infection have emphasized the necessity of prevention, early diagnosis, and successful treatment. However, diagnosing a postoperative spinal infection before clinical symptoms become apparent is extremely difficult since clinical diagnosis is primarily based on magnetic resonance imaging, which is expensive and not always available [7, 14]. Therefore, there is an urgent demand for a cheap and easy diagnostic method, which can probably be achieved through the use of biomarkers.

A variety of biomarkers have been identified as having the potential to be used to diagnose postoperative spinal infection, including C-reactive protein (CRP), white blood cell count (WBC), erythrocyte sedimentation rate (ESR), and body temperature (BT) [7, 15, 16]. Although easy to measure, the specificities of these biomarkers are not high enough to always accurately distinguish infection versus non-infection. Recent studies have identified procalcitonin (PCT) as a potential indicator for postoperative infection [14, 17]. PCT is the peptide precursor of calcitonin, a hormone that is synthesized by the parafollicular C cells of the thyroid and is involved in calcium homeostasis [18]. A comparative study involving PCT demonstrated that PCT had higher specificity than conventional markers of inflammation for indicating postoperative infection, since serum levels of PCT did not increase during the postoperative course following elective spinal surgeries of infection-free patients [14]. Although promising, the ability of PCT to distinguish patients with a postoperative infection (PI) from those with no postoperative infection (PN) has not been quantified and evaluated.

Besides PCT, soluble CD14 subtype (sCD14-ST), the soluble form of a glycoprotein expressed on the surface membranes of monocytes and macrophages, could also be a promising biomarker. sCD14-ST is released into the circulation through proteolytic cleavage on stimulated monocytes upon a pro-inflammatory signal against infectious agents [19, 20]. It has been shown to increase in response to a variety of microbial infections and inflammation [21]. Moreover, serum levels of sCD14-ST are highly correlated to a variety of bacterial infections, including infections after trauma and invasive surgical procedures [20, 21]. Therefore, SCD14-ST could be a possible candidate for a sensitive diagnostic biomarker of postoperative spinal infection. However, similar to PCT, the ability of sCD14-ST to indicate a postoperative infection after spine surgery has not been quantified and evaluated.

Therefore, we demonstrated an observational study to evaluate the abilities of PCT and sCD14-ST to indicate a postoperative infection in patients after spine surgery. We hypothesized that both PCT and sCD14-ST could be good biomarkers of postoperative infection and aimed to quantify the specificity and sensitivity obtained when using PCT and sCD14-ST to diagnose postoperative infection as well as to establish perioperative reference values.

## Materials and methods

### Study design and population

We performed a retrospective single-institutional observational study that consecutively enrolled 490 (289 male/201 female) patients who underwent spinal surgery at the orthopedics at the General Hospital of Ningxia Medical University from January 2018 to December 2020. The Institutional Review Board at the General Hospital of Ningxia Medical University approved the protocol of this study. All patients were preoperatively fully informed about the study content and gave their written consent.

Exclusion criteria were as follows: less than 18 years of age; discharged less than 5 days after the spinal surgery; diagnosed with a urinary tract or respiratory tract infection before surgery; usage of antibiotics or other surgery within the 2 weeks preceding the spine surgery.

### Data collection and laboratory analysis

Clinical data relevant to the study included sex, age, body mass index (BMI), operation duration, body temperature, the number of days in hospital, site and type of surgery, and complicated diabetes. According to the Antibiotic Application Guide, all patients received preoperative antibiotic prophylaxis with cephalosporin (first generation). All patients were operated on under general anesthesia and received daily clinical examinations until discharge.

Blood samples were acquired before and 1 day after surgery. Blood samples were immediately centrifuged (3000 rpm, 10 min) and different fractions were collected. Serum samples were stored at 80 °C until analysis. Serum levels of CRP (C-reactive Protein Quantitative Detection Kit; catalog no. K1016; detection range: 0.50–200 mg/L; intra-assay CV% < 15%; inter-assay CV% < 15%), PCT (Enhanced Chemiluminescence Detection Kit; catalog no. 833,349,501; detection range: 0–20 ng/mL; intra-assay CV% < 15%; inter-assay CV% < 15%), sCD14-ST (Human sCD14-ST Presepsin ELISA Kit; catalog no. MBS3803421; detection range: 25–400 pg/ml; intra-assay CV% < 10%; inter-assay CV% < 10%; unit/price: 96 strip wells/$755) were measured according to the manufacturer’s instructions.

### Diagnosis of postoperative infection

Postoperative infection was diagnosed according to the guidelines of the Centers for Disease Control and Prevention (CDC) and evaluated from five aspects: body temperature changes, pain in the operation area, severe changes, laboratory tests, and MRI tests.

### Statistical analysis

SPSS 17.0 statistical software (version 17.0, SPSS, Inc., Chicago, IL, USA) was used for statistical analysis. Mapping analysis was performed using Graph Pad Prism 7. Count data are shown as numbers and percentages. Measurement data conforming to normal distribution were described as χ̄ ± s. Student’s *T*-test, the *F*-test, and the chi-square test were performed. Comparisons between groups were evaluated by the *χ*^2^ test. *Z*-values were calculated through the Mann–Whitney *U* test (Wilcoxon rank sum test). The Shapiro–Wilk *W* test was carried out for the normality study. Continuous variables are shown as median (interquartile range) for nonnormally distributed data. Correlations between sCD14-ST, CRP, PCT, and the operative invasiveness parameters were evaluated using the Spearman rank correlation coefficient. Predictive receiver operating characteristic (ROC) curve analysis was used to evaluate the clinical significance of PCT and SCD14-ST in the diagnosis of early infection and to establish the reference values. *P* < 0.05 was considered to show a statistically significant difference.

## Results

### Characteristics of the study population

A summary of the characteristics of the study population is shown in Table 1. Among the 490 studied cases, 34 cases (20 male/14 female, 6.94% of the total) were diagnosed with a postoperative infection. To be specific, 19 cases (11 male/8 female) were superficial and deep incision infections, accounting for 55.88% of the total postoperative infection cases; 11 cases (5 male/6 female) were lung infections, accounting for 32.55%; 2 cases (1 male/1 female) were urinary tract infections, accounting for 5.88%; and 2 cases (1 male/1 female) were skin and soft tissue infections, accounting for 5.88%.

**Table 1:** Clinical characteristics and perioperative data

Preoperative diagnosis	Number of patients (%)	Male/female
Deformity	86 (17.5)	51/35
Tumor	14 (3.6)	6/8
Degenerative	307 (62.6)	177/130
Trauma	83 (16.9)	36/47

### Risk factor analysis of postoperative spinal infection

We first performed univariate analysis based on data obtained from clinical records to identify the factors related to postoperative infection in patients. The identified significant risk factors (*P* < 0.05) included: hospital stay ≥ 15 days, operation duration ≥ 3 h, implant placement, and complications of diabetes (Table 2).

**Table 2:** Risk factors for infection after spinal surgery

Grouping criteria		No. of patients	Infected cases	Infection rate (%)	*χ*^2^	P*
Days in hospital (days)	≥ 15	335	30	8.95	8.769	0.001
	< 15	155	4	2.58		
Operation time (min)	≥ 3	363	31	8.53	7.389	0.002
	< 3	127	3	2.36		
Antibiotic use (days)	≥ 10	285	27	9.47	8.650	0.004
	< 10	205	8	3.84		
Implant placement	Y	353	30	8.49	8.843	0.002
	N	137	4	2.91		
Concurrent diabetes	Y	299	30	10.03	13.961	0.001
	N	191	4	2.09		
Indwelling catheterization	Y	480	34	7.08	0.761	0.382
	N	10	0	0		

P* < 0.05 was considered to show a statistically significant difference

### Patient serum PCT, sCD14-ST, and CRP levels

We next divided the studied cases into postoperative infection (PI) and non-infection (PN) groups based on the diagnosis of postoperative infection, and measured the serum levels of PCT, CRP, and sCD14-ST in each group. We performed a one-way analysis of variance to test the significance of the difference between the two groups (Table 3). Our results showed that serum levels of PCT, CRP, and sCD14-ST after surgery were significantly higher (*P* < 0.01) than before surgery. Serum levels of PCT, CRP, and sCD14-ST were statistically significantly higher in the PI group as compared with the PN group (*P* < 0.01).

**Table 3:** Patient serum PCT, sCD14-ST, and CRP levels

	BS	PN group	PI group	*F*	*P*
PCT (ng/ml)	0.12 ± 0.03	0.56 ± 0.07	1.46 ± 0.13	58.393	0.000
sCD14-ST (pg/ml)	123.13 ± 1.29	168.59 ± 11.38	678.39 ± 39.17	94.296	0.000
CRP (mg/ml)	2.43 ± 1.39	11.37 ± 9.27	58.12 ± 32.79	76.336	0.000

*BS* before surgery, *PI* postoperative infection, *PN* postoperative non-infection, *PCT* procalcitonin, *sCD14-ST* soluble CD14 subtype, *CRP* C-reactive protein. *F* is the result of the *F*-test; *P* is the* P*-value from the *F*-test

*P* < 0.05 was considered to show a statistically significant difference

### Correlations between serum levels of biomarkers and operation duration

**Fig. 1:**
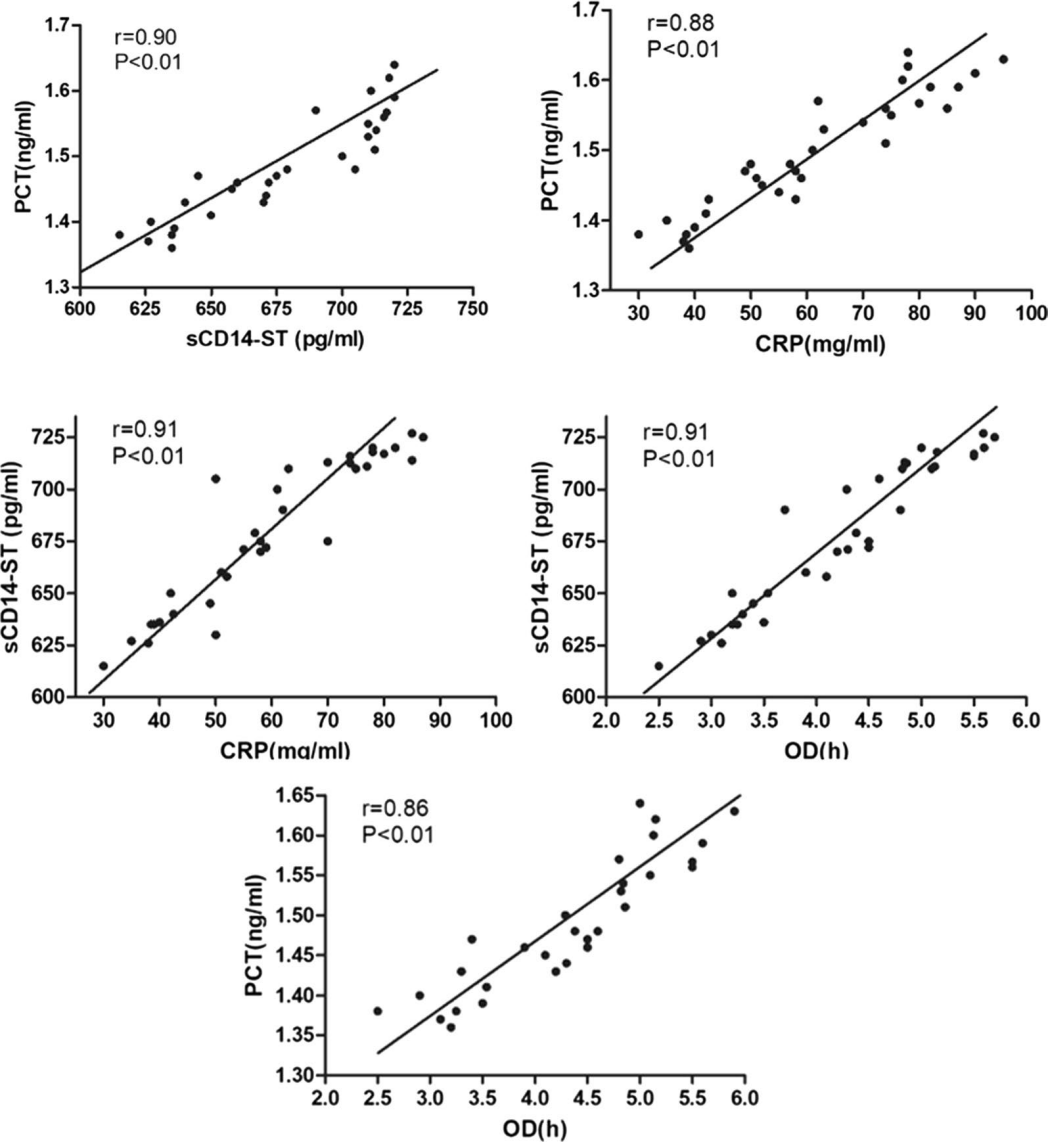
Correlations among serum levels of PCT, CRP, and sCD14-ST and OD, evaluated using the Spearman rank correlation coefficient. *P* < 0.05 was considered statistically significant. *PCT* procalcitonin, *sCD14-ST* soluble CD14 subtype, *CRP* C-reactive protein, *OD* operation duration

We then evaluated the correlations between serum levels of PCT, CRP, and sCD14-ST and operation duration (OD) in PI cases (Fig. 1). Our results showed that serum levels of PCT (*r* = 0.86; *P* < 0.01) and sCD14-ST (*r* = 0.91; *P* < 0.01) had positive correlations with OD. Serum levels of PCT, CRP, and sCD14-ST were also positively correlated with each other (PCT and CRP, *r* = 0.88; *P* < 0.01; PCT and sCD14-ST, *r* = 0.90; *P* < 0.01; CRP and sCD14-ST, *r* = 0.91; *P* < 0.01).

### Receiver operating characteristic curve analysis of PCT and sCD14-ST

**Fig. 2:**
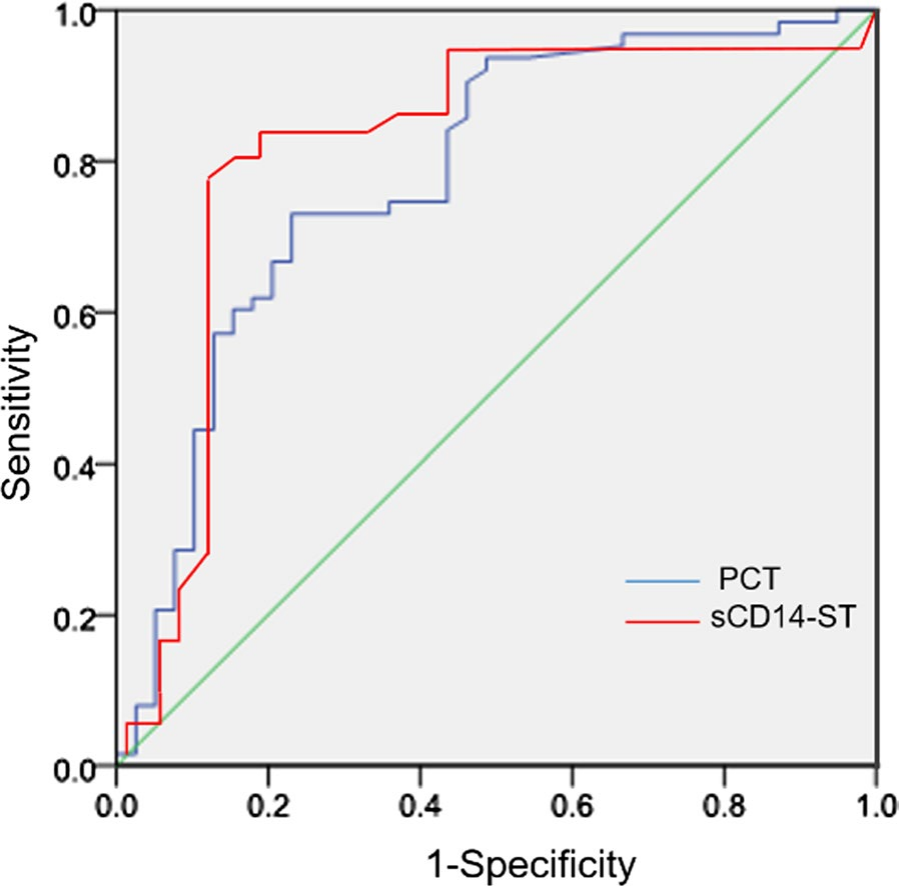
Receiver operating characteristic (ROC) curve analysis of the variations of PCT and sCD14-ST in SS patients. *P* < 0.05 was considered statistically significant. *PCT* procalcitonin, *sCD14-ST* soluble CD14 subtype

To evaluate the sensitivity and specificity obtained when using serum levels of PCT and sCD14-ST to distinguish PI versus PN, we performed receiver operating characteristic (ROC) curve analysis of the variations of serum levels of PCT and sCD14-ST in PI and PN cases and calculated the area under the ROC curve (AUC) and the optimal cutoff values (Fig. 2, Table 4). Both PCT and sCD14-ST showed high sensitivity and specificity when used to distinguish PI versus PN at the optimal cutoff value.

**Table 4:** ROC curve analysis of the variations of PCT and sCD14-ST in SS patients

Item	AUC	95% CI	Sensitivity (%)	Specificity (%)	BCV	*P*
PCT	0.817	0.80–0.93	69.3	94.9	0.69 ng/ml	0.000
sCD14-ST	0.824	0.71–0.94	71.9	91.4	258.27 pg/ml	0.00

*AUC* area under the ROC curve, *BCV* best critical value, *PCT* procalcitonin, *sCD14-ST* soluble CD14 subtype

*P* < 0.05 was considered statistically significant

### Discussion

In our study, we measured serum levels of PCT, CRP, and sCD14-ST before and 1 day after the surgical operation in 490 patients who underwent spinal surgery. We examined the correlations among the measured biomarkers and evaluated the sensitivity and specificity obtained when using PCT and sCD14-ST as diagnostic markers for postoperative infections. Our results suggested that serum levels of SCD14-ST, CRP, and PCT increased after spinal surgery, and were even higher during a postoperative infection. Serum levels of PCT, CRP, and sCD14-ST were positively correlated with each other and operation duration. Our results also suggested that PCT and sCD14-ST are potentially sensitive and specific diagnostic biomarkers for postoperative infection after spinal surgery.

Despite the rapid development of aseptic technology, postoperative infection after spinal surgery remains a devastating complication with both a prolonged detrimental impact and a frequent incidence. The traditional and most well-established diagnosis of postoperative infection after spine surgery is primarily based on magnetic resonance imaging, which is not always available [14] due to its expense and complicity. As a result, postoperative infections are only noticed when symptoms become apparent, which is past the best time for treatment. Therefore, using biomarkers as a fast and easy method to diagnose a postoperative infection in its early stages is of vital importance, and such biomarkers are in urgent demand. Although a variety of biomarkers have been identified as presenting significant changes in their serum levels during a postoperative infection after spine surgery, including C-reactive protein (CRP), white blood cell count (WBC), erythrocyte sedimentation rate (ESR), body temperature (BT), and procalcitonin (PCT) [7, 15, 16], the sensitivity and specificity of each of these biomarkers were either not examined or were not high enough to always accurately distinguish infection versus non-infection. Our study, for the first time, performed receiver operating characteristic (ROC) curve analysis to examine the sensitivity and specificity obtained when PCT and sCD14-ST are used as diagnostic markers for postoperative infection after spine surgery. Our results suggested that PCT and sCD14-ST are potentially sensitive and specific diagnostic markers, and indicated the optimal cutoff for diagnosis, which can guide further studies and the development of diagnostic methods.

PCT is the peptide precursor of calcitonin, synthesized by the parafollicular C cells of the thyroid. It functions to maintain calcium homeostasis and has been shown to be involved in a variety of infections [18, 22–24]. Our results show increased serum levels of PCT after spine surgery, and even higher serum levels of PCT in cases with a postoperative infection, which agrees with previous studies of the involvement of PCT during infection [18, 22–24], the relationship between calcium homeostasis and infection, as well as the critical role of PCT in calcium homeostasis [25, 26]. Besides, PCT is shown to positively correlate with operation duration, which agrees with a previous discovery that postoperative infection correlates with operation duration [27]. In addition, our ROC analysis shows that PCT has high sensitivity and specificity when used to diagnose postoperative infection with the optimal cutoff of 0.69 ng/ml, indicating that diagnostic approaches based on PCT can be developed.

Unlike PCT, sCD14-ST had never been identified as a biomarker for postoperative infection after spine surgery until our study. sCD14-ST is the soluble form of cluster of differentiation 14 (CD14), which is well known as a cell-surface receptor for complexes of lipopolysaccharide and its binding protein, which is present in macrophages, monocytes, and granulocytes [25, 26]. It has been found to increase in patients with sepsis [28]. SCD14-ST is produced in response to bacterial infection and is highly correlated with a variety of immune and inflammatory responses. It starts to rise 2 h after inflammation and reaches its peak in 3 h [20, 28]. Our results, for the first time, indicate the potential of using sCD14-ST as a sensitive and specific diagnostic biomarker. sCD14-ST was shown to increase after spine surgery and during a postoperative infection, which can be explained by the function of sCD14-ST in immune and inflammation responses [29, 30]. Similar to PCT, sCD14-ST positively correlates with operation duration and also has high sensitivity and specificity when used to diagnose postoperative infection using with the optimal cutoff of 258.27 pg/ml. Besides, the serum level of sCD14-ST highly positively correlates with PCT, suggesting that both may be used together to diagnose postoperative infection with increased confidence. Based on our data, we suggest including both markers (PCT and sCD14-ST) in early postoperative routine blood checks on patients, even when they do not show clinical signs of infection. If abnormal values are observed, actions should be taken to prevent the potential infection. These actions include, but are not limited to, performing more frequent routine inspections such as blood checks and using preventive antibiotics.

Although our study demonstrated two promising sensitive and specific diagnostic markers for postoperative infection after spine surgery, certain limitations of this study cannot be ignored. (1) All cases involved in our study are from the Ningxia area, which may not be enough to represent a global trend. (2) All cases involved in our study are patients who underwent spinal surgery at the Department of Orthopedics in the General Hospital of Ningxia Medical University. The incidence of postoperative infection may be influenced by the aseptic operations performed by this specific hospital. (3) Our study only focuses on PCT and sCD14-ST, based on previous studies. A comprehensive analysis involving a large number of potential biomarkers may still be in demand to develop diagnostic approaches. (4) Our study only measured PCT and sCD14-ST before and 1 day after surgery; subsequent measurements may also be needed to evaluate the usage of PCT and sCD14-ST as diagnostic markers.

## Conclusion

In conclusion, our study demonstrated that serum levels of PCT and sCD14-ST could be used as sensitive and specific diagnostic biomarkers for postoperative infection after spine surgery, providing new ideas for diagnosing infection at an early stage.

## Data Availability

The data that support the findings of this study are available from the corresponding author, Jiandang Shi, upon reasonable request.

## Abbreviations

PCT: Procalcitonin
sCD14-ST: Soluble CD14 subtype
SS: Spinal surgery
BS: Before surgery
PI: Postoperative infection
PN: Postoperative non-infection
ROC: Receiver operating characteristic
CRP: C-reactive protein
